# Is the ASA Score in Geriatric Hip Fractures a Predictive Factor for Complications and Readmission?

**DOI:** 10.1155/2016/7096245

**Published:** 2016-05-12

**Authors:** G. Kastanis, A. Topalidou, K. Alpantaki, M. Rosiadis, K. Balalis

**Affiliations:** Department of Orthopaedics and Traumatology, University Hospital of Heraklion, Faculty of Medicine, University of Crete, Crete, Greece

## Abstract

Hip fractures are the second cause of hospitalization in geriatric patients. The American Society of Anesthesiologists (ASA) classification scheme is a scoring system for the evaluation of the patients' health and comorbidities before an operative procedure. The purpose of this study was to determine whether the ASA score is a predictive factor for perioperative and postoperative complications and a cause of readmission of geriatric patients with hip fractures. The study included 198 elderly patients. The mean values of hospitalization were 6.4 ± 2.1 days for the patients with ASA II, 10.4 ± 3.4 days for the patients with ASA III, and 13.5 ± 4.4 days for the patients with ASA IV. The patients with ASA II exhibited minor complications, while patients with ASA III presented cutaneous ulcer and respiratory dysfunction. Five patients with ASA IV had pulmonary embolism, two patients had myocardial infarction, and three patients died. The ASA score seems to have direct correlation with multiple factors, such as the hospitalization days, the severity of the complications, and the total hospitalization costs. The treatment of geriatrics hip fractures in patients with a high ASA score requires a multidisciplinary approach and a special assessment in order to decrease postoperative morbidity and mortality and offer optimal functionality.

## 1. Introduction

Hip fractures are a common and serious injury in elderly patients and they constitute the second cause of hospitalization [1]. The majority of hip fractures in elderly population mark the beginning of a downward trend in the patients' health. More than 1.6 million hip fractures occur worldwide each year. On average, hip fractures reduce life expectancy by 25% in comparison with the age-matched general population. In addition, hip fractures are linked to the high cost that is associated with the care of these patients and burdens on the health care systems [2].

The main treatment goal for these injuries is early mobilization in order to prevent complications that are associated with prolonged immobilization. Another important goal is the return to prefracture functional activity, which can be achieved with surgery. Unfortunately, the return to an optimal functional level after a hip surgery is not determined by the type of operation but by the preoperative comorbidities and the postoperative complications [3]. The mortality rates of geriatric hip fractures in one year vary from 18% to 33% [4]. This demonstrates the need to determine and restrict the major postoperative complications that lead to these high rates of mortality.

The American Society of Anesthesiologists (ASA) physical status classification system was introduced in 1941 by Meyer Saklad, Emery Rovenstine, and Ivan Taylor as a grading system for the surgical patients' preoperative health [5, 6]. In 1963, the ASA suggested a revised classification regarding the physical status of preoperative patients reducing the number of classes from seven to five [7]. The correlation between the ASA scores and the rate of postoperative complications has been reported in the literature [8, 9]. Several studies indicate the close relation of the ASA score not only to morbidity and mortality but also to the operating time, the hospitalization duration, the surgery duration, the postoperative infections, the health care costs, and many more variables [10–16]. Specifically, it is known that the ASA classification is a good predictor of morbidity, mortality, complications, and medical problems both in the perioperative and in the postoperative period that follows hip fracture surgery in elderly patients. However, whether and how the ASA classification is related to the functionality and the postoperative restoration of mobility are unclear and need further investigation [4, 9, 12, 13, 16].

The purpose of this prospective study was the evaluation of the prognostic value of the ASA score as a strong predictive factor for postoperative complications and hospital readmission in geriatric patients with hip fractures. In addition, the relationship between the prefracture comorbidities (hypertension, congestive heart disorders), the development of postoperative complications (cardiac, pulmonary, neurological, and surgical complications), the need for prolonged postoperative hospitalization, and the restoration of functionality depending on the patients' answers and satisfaction were investigated.

## 2. Material and Methods

### 2.1. Population and Data Collection

All patients, who were ≥65 years old with an isolated acute hip fracture and were admitted to the General Hospital of Sitia between January 2009 and December 2012, were enrolled in this prospective, observational cohort study. The patients with pathological fractures (such as metastatic fractures), malignant diseases, fractures due to Paget's disease, and inability to walk before the fracture, the patients who had received nonoperative treatment, and the patients who were younger than 65 years old were excluded. Also, two patients with missing data due to no information about their accurate age and an unclear medical history for one of them were excluded. The population of this study came from a geographically defined area.

All data, such as patient demographics, the ASA physical status classification, the functional status, the type of fracture, the complications and comorbidities, the days from the hospital admission to the surgery, the type of operation, the type of anesthesia, the hospital discharge, and the readmission in 30 days, was extracted from the medical records and collected into a database. All data was saved with a serial number and no personal identifiers were used. The preoperative examinations in all patients included X-rays, an electrocardiography, and blood tests. The ASA score was assigned just before the operation as part of the anesthesia preoperative examination. The postoperative attention was focused on pulmonary disorders, significant arrhythmias, urinary tract infections, and wound inflammations.

Based on the treatment protocol, all patients were encouraged to stand up with support and partial weight bearing as tolerated from the first postoperative day. The type of the operation was based on the type of fracture.

The patients' satisfaction and functionality were assessed postoperatively after three to six months. The patients were contacted by telephone or they visited the outpatient orthopaedic clinic. They were asked to answer whether they considered that they returned to their preoperative functional status and whether they were satisfied with the postoperative result. If both answers were negative, the patients were classified as the “poor functionality-satisfaction” group. If even one was positive, the patients were classified as the “moderate functionality-satisfaction” group. Finally, if both answers were positive, the patients were classified as the “good functionality-satisfaction” group. A five-point scale or another scale was avoided because it seemed to be difficult to understand or it confused some participants.

### 2.2. Statistical Analysis

All analyses were carried out with the SPSS® statistical package, version 15.0 (SPSS Inc., Chicago, IL, USA) for Windows. All statistical tests were carried out at the 5% level of significance and were two-sided. Descriptive statistics, including frequencies, means, and standard deviations (SD) were computed. The data normality was assessed with the Kolmogorov-Smirnov test. Depending on the data distribution, the independent samples *t*-test, the paired *t*-test, and the Analysis of Variance (ANOVA) (with post hoc Bonferroni adjusted tests) were used to compare groups of continuous data with a 95% confidence interval (CI). The ordinal data were compared by the chi-square test. The correlations between the variables and the continuous data were assessed with Pearson's *r*. The ASA II, ASA III, and ASA IV patients were classified into three groups. The patients' answers regarding functionality and satisfaction were classified as “1 = yes” and “2 = no.”

## 3. Results

The demographic characteristics of the participants (*n* = 198), the cause of injury, the type of fracture, the ASA score, the type of anesthesia, the average waiting time for surgery, and the average time of hospitalization are presented in Table 1.

One hundred and seven fractures were extra-articular and 91 were intracapsular. The treatment involved fracture osteosynthesis in 116 cases and prosthetic replacement in 82 cases.

The females had statistically significantly higher mean age than men (*p* < 0.001, CI 95%,  −9.86, and −5.01). However, there was no statistically significant difference in the Body Mass Index (BMI) concerning the gender.

The ASA score was statistically significantly higher for men compared with women (*p* = 0.019, CI 95%, –0.44, and –0.03). There was no statistically significant difference (*p* = 0.772) in the waiting time for surgery among patients with ASA II and patients with ASA III. However, a statistically significant difference appears in the waiting time among patients with ASA II (*p* < 0.001, CI 95%, –2.05, and –4.32) and patients with ASA III (*p* < 0.001, CI 95%, –3.66, and –3.90) compared with the patients with ASA IV. Regarding the average time of hospitalization, there were statistically significant differences among all groups that were classified with ASA score. More specifically, the patients with ASA II had statistically significantly shorter hospital stay compared with the patients with ASA III (*p* = 0.005, CI 95%, –0.42, and –3.01) and patients with ASA IV (*p* < 0.001, CI 95%, –2.67, and –6.22). Also, the patients with ASA III had statistically significantly shorter hospitalization compared with the patients with ASA IV (*p* = 0.001, CI 95%, –0.98, and –4.48).

Twelve different medical comorbidities and eleven complications were reported in the patients' medical records. Any condition that required treatment by a physician, who was trained to handle it appropriately, was considered as a medical complication. All comorbidities and postoperative complications that are categorized with ASA score are presented in Table 2. It is worth mentioning that the patients with ASA II exhibited only wound and urinary infection, while the patients with ASA III and the patients with ASA IV presented several complications. The most common complications in the patients with ASA III were cutaneous ulcer (7.7%) and congestive heart failure (5.5%) and in the patients with ASA IV were cutaneous ulcer (19.4%), pneumonia (19.4%), urinary infection (16.1%), and pulmonary embolism (16.1%). In addition, the assessment of the dependence relationship between the ASA score and the complications exhibited a very strong linear correlation (*r* = 0.778, *p* < 0.001). The sum of the complications that occurred in each group was classified with ASA score and is presented in Table 2. The most common comorbidities in all patients despite the ASA score were hypertension and fluid-electrolyte disorders. There were three deaths in all cases (1.5%) and all concerned patients with ASA IV. This means that the mortality of patients with ASA IV was 3.2%. This mortality rate refers only to cases during hospitalization.

Moreover, no patient with ASA II was transferred to another unit or needed rehospitalization. On the other hand, *n* = 17 (18.7%) patients with ASA III and *n* = 19 (61.3%) patients with ASA VI were transferred to other units. Also, *n* = 11 (12.1%) patients with ASA III and *n* = 8 (25.8%) patients with ASA VI needed rehospitalization.

Finally, regarding the condition of functionality and the total satisfaction (functionality-satisfaction), there was no linear correlation. The patients with ASA II had statistically significantly greater outcomes (*p* = 0.015, CI 95%, 0.06, and 0.8) compared with the patients with ASA IV. There were no statistically significant differences among patients with ASA III and patients with ASA II and ASA IV.

## 4. Discussion

More than 85% of geriatric patients with hip fractures have their hospitalization services covered by national medical care systems. It is estimated that 458,000 to 1,037,000 hip fracture incidents will occur in the USA by the year 2050. In a period of fifty years, between 2000 and 2050, an increase of 135% is predicted in the number of older people. This leads to an increase in the percentage of geriatric hip fractures and subsequently an increase in the growth of health care costs. Moreover, the total cost is burdened with other factors, such as longer recovery periods, postoperative complications and comorbidities, and prolonged hospital stay [17–20].

Based on the abovementioned information, the need for a risk-adjusted reimbursement system arises. The US government uses the ASA classification system as a risk-adjustment tool, which identifies the patients' factors that help predict the hospitalization costs [20, 21]. In addition, apart from the cost-morbidity correlation, the overall patients' health status and their postoperative condition constitute important factors.

Recent studies showed that not only is the ASA score correlated with multiple factors that increase the surgical resource utilization, including infections [22], reoperations [23], intraoperative blood loss [24], the hospitalization duration, and the postoperative complications and comorbidities which would require diagnostic and imaging investigation [7, 9, 12, 22] but it is also a strong predictor of the postoperative outcomes [10].

This study showed that women had higher age average and men exhibited a greater ASA score, results that are in agreement with the findings of other researchers [16, 25, 26]. This agreement cannot be explained because gender is not an isolated factor as a variable and possibly more specific research in this field is required. Also, it is reported that men have more postoperative complications [27], possibly due to their preoperative health status.

Regarding the ASA score, the patients who were classified as ASA IV had a longer waiting time for surgery. However, this relationship is bidirectional since it is known that surgery delay doubles the risk of major complications and postoperative morbidity [28]. Therefore, the patients with ASA IV and ASA III should receive specialized medical care so that the perioperative and postoperative complications could be addressed and possibly avoided [4]. At the same time, the direct proportional relationship between the ASA score and the total hospitalization duration (greater ASA score results in longer hospitalization) demonstrates the need for an algorithmic reimbursement model based on the ASA score. This would optimize the relationship between health services and hospitalization costs [13, 20, 23].

Initially, the ASA classification system was created by anesthesiologists to estimate the risk of operative morbidity based on the patients' health status [6]. Nevertheless, this study, as well as others, showed that the ASA score is strongly correlated with postoperative comorbidities and complications, which means that the ASA score can also estimate postoperative morbidity. The higher the ASA score is, the greater the percentage of postoperative complications and medical interventions is [4, 9, 10, 28]. At the same time, mortality and the need for transfer to other units are proportionally correlated with the ASA score. Therefore, as a result of the abovementioned information, the total cost of health services is growing along with the increase in the ASA score.

However, financial studies indicate that the most expensive part of the total cost is surgery, composing 72–88% of the total cost. The intraoperative costs can be further increased if one considers the greater blood loss, the surgery duration, and other factors which are proportionally dependent on the patients' health status. Therefore, surgery and surgical services can be potential areas of improvement which can change interrelated higher percentages of postoperative complications and health care costs [10, 13, 20]. Also, in geriatric hip fractures the multidisciplinary approach and treatment, the early investigation of high risk patients, and the daily individualized care resulted in fewer postoperative complications and transfers to other units, better ambulatory status, and decreased length of stay [4, 19, 28, 29].

Regarding the postoperative functionality and satisfaction, the patients with an increased risk prior to the surgery were hardly restored to a satisfactory functional life. However, further investigation into this field is needed [9, 30]. It would also be important to investigate the influence of the psychological-mental status and the physical status separately.

The limitation of this study was the relatively small number of patients and the absence of follow-up. However, the results are valid and important as they exhibit many interrelated factors associated with the health progress of a geriatric patient.

## 5. Conclusion

The ASA classification system is correlated with multiple factors, which can lead to the prediction of the postoperative status. The patients with ASA III and ASA IV should receive individual treatment, taking into account their entire preoperative health profile and avoiding the surgery delay. Also, the physicians should focus more on restoring the functionality of these patients.

## Figures and Tables

**Table 1:** Characteristics of hip fracture cohort (*n* = 198).

Characteristics	Mean (SD) or number of patients	%
Demographics		
Age	85.4 ± 9	
BMI	23.9 ± 3.6	
Female	143	72.2%
Male	55	27.8%

Cause of injury (number of patients)		
Fall from standing height	106	53.5%
Fall downstairs or off a step ladder	81	40.9%
Vehicle accident	11	5.6%

Type of fracture (number of patients)		
Subcapital	91	45.6%
Intertrochanteric	87	43.9%
Subtrochanteric	20	10.1%

ASA score (number of patients)		
II	76	38.4%
III	91	45.6%
IV	31	15.7%

Average waiting time for surgery categorized by the ASA score (number of days)		
II	2.3 ± 1.2	
III	5.2 ± 1.1	
IV	8.4 ± 2.9	

Average time of hospitalization categorized by the ASA score (number of days)		
II	6.4 ± 2.1	
III	10.4 ± 3	
IV	13.5 ± 4.4	

Type of anesthesia		
Epidural	187	94.4%
General	11	5.6%

**Table 2:** Comorbidity profile and complications of hip fracture cohort categorized by the ASA score. Many patients have more than one comorbidity or complication.

Conditions/complications	ASA II (*n* = 76)		ASA III (*n* = 91)		ASA IV (*n* = 31)	
	*n*	%	*n*	%	*n*	%
Cutaneous ulcer			7	7.7%	6	19.4%
Wound infection	5	6.6%	4	4.4%	2	6.5%
Pneumonia			4	4.4%	6	19.4%
Pulmonary embolism			2	2.2%	5	16.1%
Congestive heart failure			5	5.5%	4	12.9%
Cerebrovascular accident			3	3.3%	2	6.5%
Acute renal failure			2	2.2%	2	6.5%
Ileus			1	1.1%	1	3.2%
Myocardial infarction					2	6.5%
Urinary infection	4	5.3%	3	3.3%	5	16.1%
Death					3	9.7%
*Total number of complications that occurred*	*9*		*31*		*38*	

Comorbidities	ASA II (*n* = 76)		ASA III (*n* = 91)		ASA IV (*n* = 31)	
	*n*	%	*n*	%	*n*	%

Hypertension	51	67.1%	76	83.5%	30	96.8%
Deficiency anaemia	17	22.4%	29	31.9%	8	25.8%
Fluid and electrolyte disorders	18	23.7%	31	34.1%	12	38.7%
Chronic pulmonary disease	14	18.4%	33	36.3%	11	35.5%
Diabetes mellitus	7	9.2%	8	8.8%	7	22.6%
Neurological disorders	9	11.8%	9	9.9%	6	19.4%
Thyroidism	13	17.1%	18	19.8%	6	19.4%
Congestive heart failure	10	13.2%	12	13.2%	5	16.1%
Depression	5	6.6%	9	9.9%	4	12.9%
Renal failure	16	21.1%	30	33.0%	12	38.7%
Peripheral vascular disorder	12	15.8%	17	18.7%	16	51.6%
Solid tumor	0	0%	1	1.1%	1	3.2%
